# Sichuan dark tea improves lipid metabolism and prevents aortic lipid deposition in diet-induced atherosclerosis model rats

**DOI:** 10.3389/fnut.2022.1014883

**Published:** 2022-11-24

**Authors:** Rui Lu, Takumi Sugimoto, Tomoe Tsuboi, Tatsushi Sekikawa, Mamoru Tanaka, Xiaohua Lyu, Shinji Yokoyama

**Affiliations:** ^1^Food and Nutritional Sciences, Chubu University, Kasugai, Japan; ^2^Department of Nutrition and Food Hygiene, West China School of Public Health and West China Fourth Hospital, Sichuan University, Chengdu, China

**Keywords:** atherosclerosis, dark tea, HDL, triglyceride, ABCA1, lipases

## Abstract

**Background and aims:**

Sichuan dark tea (ST), Zangcha, is a traditional fermented Chinese tea found in Sichuan and Tibet and claimed for beneficial effects against lifestyle-related metabolic disorders. We examined the effects of ST on lipid metabolism and atherosclerosis.

**Methods and results:**

Sichuan dark tea was given to fat-rich diet-induced atherosclerosis model rats in comparison with dark-fermented Chinese Pu-erh tea (PT) and Japanese green tea (GT). After 8 weeks of feeding, ST and PT induced an increase in high-density lipoprotein (HDL)-cholesterol and a decrease in glucose, and ST decreased triglyceride in plasma. ST also induced low pH in the cecum. There was no significant change in their body weight among the fat-feeding groups but a decrease was found in the visceral fat and liver weight in the ST group. Accordingly, ST reduced lipid deposition in the aorta in comparison with PT and GT. ST increased mRNA of LXRα, PPARα, PPARγ, and ABCA1 in the rat liver. The extract of ST stimulated the AMPK pathway to increase the expression of ABCA1 in J774 cells and increased expression of lipoprotein lipase and hormone-sensitive lipase in 3T3L1 cells, consistent with its anti-atherogenic effects in rats. High-performance liquid chromatography analysis showed unique spectra of original specific compounds of caffeine and catechins in each tea extract, but none of them was likely responsible for these effects.

**Conclusion:**

Sichuan dark tea increases plasma HDL and reduces plasma triglyceride to decrease atherosclerosis through AMPK activation. Further study is required to identify specific components for the effects of this tea preparation.

## Introduction

Tea is a long-time favored beverage in the history of human culture and is believed to have various beneficial effects on human health. It is produced from the leaves of *Camellia sinensis* originating in China (*C. sinensis var. sinensis*) and India (*C. sinensis var. assamica*). The tea products in China and Japan are all made from the former. Tea is taken as various preparations largely classified into two categories of straight green tea (GT) and its fermented products. Dark-fermented tea is one of the latter popular in China and has empirically been claimed for its beneficial effects on health in tradition. It has therefore become a subject of interest and investigation in health and nutritional sciences, and the evidence has accumulated for the background of the effects against various lifestyle-related metabolic disorders, such as obesity, diabetes, hypertension, and atherosclerosis. Most of these works were carried out for popular and commonly consumed Pu-erh tea (PT) but some were for other types of dark teas as well (1–6). Preliminary findings indicated the activation of peroxisome proliferator-activated receptors (PPARs), liver X receptors (LXRs), and farnesoid X receptors (FXRs) by some fermentation-processed teas (7, 8), and the topic was well summarized in an extensive review article by Zhang (9). On the other hand, the risk of toxicity of PT was also discussed in general for the accumulation of fluoride in old tea leaves and the production of aflatoxins during fermentation (10).

Sichuan dark tea (ST), Zangcha, is one of the traditional dark-fermented teas commonly consumed in the regions of Sichuan and Tibet in China (11). The historical background of this tea is that it originates in the plants in Yunnan Province and their rough old leaves are fermented during long transportation to Tibet through Sichuan. In these days, it is mainly produced in Ya’an area of Sichuan Province, located in the middle of the historic long trail from Yunnan to Tibet. This is a unique and local type of fermented tea that has come down in history with a strong local belief of its beneficial effects on health. Because it has been less popular even among the Chinese, scientific investigation has not been done much in comparison with PT (12–14). Recently, Li et al. have reported the effects of an ST-based formula designed on the idea of “medicine-food homology” (15). The formula reduced triglyceride and raised HDL-cholesterol in the plasma of apolipoprotein E-deficient mice, in association with changes in the intestinal microbial flora (15). The results indicated its potential preventive effect against atherosclerosis development. It is therefore important to investigate this particular dark-fermented Chinese tea in further detail.

Based on these backgrounds, we studied the effects of ST on atherosclerosis development in the fat-rich diet-induced rat model. We chose Chinese PT and Japanese GT, as references, since these were more common types of tea for the previous investigation as described above. It was shown that ST has more specific effects than other types of tea on lipid metabolism and atherosclerosis development in a specific animal model used in this study.

## Materials and methods

### Tea products and their processing

Three types of tea products were subjected to investigation, representing dark-fermented, and fresh green teas. ST under the product name Zangcha was provided by Guge Tibetan Tea Culture Communication Co., Ltd., in Ya’an, Sichuan, China. Pu-erh tea (PT) was a commercial product of Yunnan Province, China and purchased in Japan through China Trading Co., Ltd., Yokohama. Deep-steamed GT was a commercial product of ITO EN, Tokyo, Japan. For animal experiments, the dry leaves of 20 g were boiled in 1 liter water at 100°C for 45 min using a heat insulation tank (Shaking Bath BW 101, Yamato Kagaku Co., Ltd., Tokyo), cooled to room temperature, and filtered before feeding. For the experiments with cultured cells, the tea extracts were prepared by boiling 20 g of tea leaf in 300 mL water for 45 min followed by filtering and drying with a centrifugal evaporator (CVE-3110 type, EYELA Tokyo Rika Kikai Co., Ltd., Tokyo) at 30°C, 900 rpm and −100 kPa for 45 h as designed based on previous reports (16, 17). The extracts were stocked in a solution of 1 mg/mL with distilled water. For the analysis by high-performance liquid chromatography (HPLC), the tea leaf extract of 2 g boiled in 100 mL water for 45 min was filtered for 0.22 μm and diluted twofold with distilled water.

### Animal experiments

Six-week-old male Sprague–Dawley rats were divided into five groups (*n* = 9 in each) and housed under alternating 12-h light/dark cycles with a relative humidity of 60 ± 10% and a controlled temperature of 22 ± 2°C. The control group was fed a control chow with 5.4% fat in calories (CRF-1, Oriental Yeast, Tokyo) and the other four groups were given a high-fat diet with 16.5% in fat (F2HFD1, Oriental Yeast) for 8 weeks (Table 1). The fat contents of these diets are substantially lower than those used in other previous experimental protocols for rats, such as 13% for control and 45–60% for high fat (18–20), reflecting the nutritional background of Japanese. The control group and plain high-fat diet group (HF) were fed with water, and the experimental groups were fed with the three types of tea (such as ST, PT, and GT) instead of water on top of the high-fat diet, as they were prepared as described above. The tea or water was taken freely as a liquid. The food and liquid intakes were measured three times a week and the body weight was measured once a week for monitoring. At the end of the experiment, blood plasma levels of total cholesterol (TC), HDL-cholesterol (HDL-C), triglyceride (TG), and glucose were measured under the condition of free feeding, by using enzymatic assay kits for lipid parameters provided by Sekisui Medical Co., Ltd., Tokyo and by Glucose CII Test Wako (GOD method, Wako Pure Chemical Industries, Ltd., Tokyo) for blood glucose level. The presence of apolipoprotein E-containing HDL in rat plasma may not likely influence the HDL assay significantly (21) but the assay system was validated for linearity and selectivity for rat plasma. Non-HDL-cholesterol (non-HDL-C) was calculated as [TC]–[HDL-C]. After the animals were euthanized under isoflurane gas, the weight of the liver and that of omentum/perirenal visceral fat was measured and expression of mRNA in the liver was estimated for LXRα, PPARα, PPARγ, ATP binding cassette transporter (ABC)A1, ABCG1, lipoprotein lipase, and hepatic lipase as described in detail below. The contents of the cecum were taken out by a spatula with saline for measuring its pH. The deposition of lipid on the inner wall of the aorta was examined by staining with Oil red O (MUTO PURE CHEMICALS Co., Ltd., Tokyo). The area of the region stained above a threshold density was quantitated by using the Photoshop software, as a relative area of a color-selected region (red) to a focused region (total surface) with a histogram tool as described (22). The protocol for the animal experiments had been approved as 2910063 by the Animal Experiments and Welfare Committee of Chubu University in accordance with its guidelines.

**TABLE 1:** Basic parameters for the animal experiment.

		C	High fat (HF)			
			
		Water	Water	ST	PT	GT
Chow composition	kcal/g	3.57	4.14			
	Carbohydrate, %	55.3	45.1			
	Protein, %	21.9	22.6			
	Fat, %	5.4	16.5			
	Cholic acid, %	0	0.25			
Food intake	Total chaw, g	1412 ± 91	1427 ± 125	1456 ± 131	1560 ± 131	1440 ± 101
	Energy, kcal	5041 ± 324	5908 ± 519	6029 ± 542	6458 ± 544	5962 ± 417
	Carbohydrate, g	781.0 ± 50.2	643.6 ± 56.5	656.8 ± 59.1	703.6 ± 59.3	649.5 ± 45.5
	Protein, g	309.3 ± 19.9	322.5 ± 28.3	329.1 ± 29.6	352.6 ± 21.7	325.5 ± 22.8
	Fat, g	76.3 ± 4.9	235.5 ± 20.7	240.3 ± 21.6	257.4 ± 21.7	237.6 ± 16.6
	Water/Tea, ml	2088 ± 248	2252 ± 254	2050 ± 254	2262 ± 275	2281 ± 303

Male rats were divided in to 5 groups (*n* = 9 each) and fed with control chow (C; CRF-1), high-fat chow (HF; F2HFD1), and Sichuan tea, Pu-erh tea, and Green tea instead of water on top of HF (ST, PT, and GT, respectively). Chow composition is in weight percent. Food intake is total amount during the 8-week experiment period.

### The cells and their culture conditions

The effects of tea extracts were examined in cell line cells modeling macrophages and adipocytes to investigate their background mechanism in preventing atherogenesis and visceral adipogenesis. Mouse monocyte-macrophage cell line cells J774.1 were maintained in RPMI 1640 medium (Sigma-Aldrich, St. Louis) containing 10% fetal bovine serum and 0.05 mg/mL gentamicin solution (Sigma-Aldrich) in a humidified atmosphere of 5% CO2 and 95% air at 37°C. The cells were seeded in culture plates at a density of 3 × 106 cells and cultured for 1 day before use. Mouse immortalized 3T3-L1 fibroblasts were cultured with DMEM medium (Sigma-Aldrich) supplemented with 10% fetal bovine serum in a humidified atmosphere of 5% CO2 and 95% air at 37°C. The cells were differentiated to adipose 2 days after reaching 80% confluence (day 0), by supplementing growth media containing 10 μg/mL insulin (Sigma-Aldrich), 1 μM dexamethasone (Wako), and 0.5 mM 1-methyl-3-isobutyl-xanthine (Sigma-Aldrich) and maintained for 4 to 8 days in growth media containing 1 μg/mL insulin. The effects of the tea extracts were examined on J774 cells and 3T3-L1 cells. The tea extract prepared as described above was added to the cells in culture in a final concentration of 1 and 5 μg/mL for 1/100 and 1/20 of the tea preparation for oral intake. J774.1 cells at an 80% confluent stage were washed with phosphate-buffered saline and treated with various concentrations of the tea extracts in RPMI 1640 medium supplemented with 0.1% bovine serum albumin (Sigma-Aldrich) and 0.05 mg/mL gentamicin solution (Sigma-Aldrich) for 18 h. After differentiation into adipocytes, 3T3-L1 fibroblasts were incubated with various concentrations of the tea extracts in the DMEM medium supplemented with 0.1% bovine serum albumin for 18 h.

### Estimation of mRNA expression by real-time quantitative polymerase chain reaction

Total cellular RNA was isolated from frozen livers or tea extracts treated cells using ISOGEN reagent (Nippon Gene, Tokyo). RNA was reverse transcribed to cDNA using iScript™ cDNA Synthesis Kit (BIO-RAD, Hercules). Gene expression was analyzed by real-time quantitative polymerase chain reactions using SYBR PCR Permix Ex Taq Kit (TaKaRa, Kyoto) on a 7300 Real-Time PCR System (Applied Biosystems, Waltham). Primer sequences used in the study are shown as follows: abca1, 5′-AAC AGT TTG TGG CCC TTT TG-3′ and 5′-GAT GAG CCA GAC TTC TGT TGC-3′ for J774.1 cells, 5′-GAA CTG GCT GTG TTC CAT TGA T-3′ and 5′-GAT GAG CCA GAC TTC TGT TGC-3′ for mouse liver, abcg1, 5′-ACG CAG TTC TGC ATC CTC TT-3′ and 5′-CGG AGT TGC TCA AGA CCT TC-3′, lxrα, 5′-TCT GGA GAC ATC TCG GAG GTA-3′ and 5′-GGC TCA CCA GTT TCA TTA GCA-3′, pparα, 5′-GGA CCT TCG GCA GCT GGT-3′ and 5′-TCG GAC TCG GTC TTC TTG ATG-3′, pparγ, 5′-ATA AAG CAT CAG GCT TCC ACT-3′and 5′-GCA CTT CTG AAA CCG ACA GTA-3′, lpl (lipoprotein lipase), 5′-GGG CAT GTT GAC ATT TAC CC-3′ and 5′-GCT GGT CCA CAT CTC AAG T-3′, hl (hepatic lipase), 5′-GAA ACC AGA GCC ATT TGG AA-3′ and 5′-AAT CTG ACA GCC CTG ATT GG-3′, and hsl (hormone-sensitive lipase), 5′-GCA CTA CAA ACG CAA CGA GA-3′ and 5′-TGT GAT CCG CTC AAA CTC GA-3′.

### Phosphorylation of 5′ adenosine monophosphate-activated protein kinase

The effect of tea extracts was examined for phosphorylation of AMPK (23). J774.1 cells were preincubated with or without dorsomorphin (DOR), an inhibitor of AMPK (predissolved in DMSO and added to make the final concentration 5 μM), for 1 h. After this treatment, the cells were incubated with or without 1 μg/mL of tea extract or 0.15 mM cAMP, an activator of AMPK, for 1 h. Cells were immediately rinsed with phosphate-buffered saline at 4°C and incubated for 5 min with gentle shaking in Lysis-M reagent (complete Lysis-M, Roche, Basel) containing complete Mini tablets (Complete Mini Protease Inhibitor Cocktail tablets, Roche) and phosphatase inhibitor tablets (PhosSTOP, Roche). The insoluble residue was removed by centrifugation at 15,000 × *g* for 10 min at 4°C to collect the supernatant as cell lysate. An equal amount of protein in the lysis buffer [50 mM Tris–HCl buffer, pH 7.5, containing 150 mM NaCl, 1% Triton X-100, 1% sodium dodecyl sulfate (SDS), and 10 mM 2Na-EDTA] in the presence of Protease Inhibitor Cocktail (Sigma-Aldrich) was analyzed in SDS-polyacrylamide electrophoresis and immunoblotting by using the monoclonal antibodies against AMPK and phosphorylated AMPK (Invitrogen) and that against ABCA1 peptide following a common sequence of its C-terminus of human and mouse CNFAKDQSDDDHLKDLSLHKN (MABI98-7, MAB Institute, Yokohama, Japan) (24), and by using that against β-actin (A5316, SIGMA) as a reference. The signals were visualized with a chemiluminescence method using ImmunoStar LD or Zeta (Wako) on Image Studio Digits (LI-COR Biosciences, Lincoln).

### Analysis of tea extracts by high performance liquid chromatography

An HPLC system for the determination of tea extracts consisted of an 1100 series system (Agilent Technologies, Santa Clara, CA, USA) with a TSKgel ODS-80Ts column (4.6 mm × 250 mm, Tosoh Co., Kyoto, Japan) and an SPD-6AV UV-VIS spectrophotometric detector (Shimadzu Co., Kyoto, Japan) using detection wavelengths at 280 nm. A two-solvent gradient elution using 10 mM KH2PO4 (A) and acetonitrile (B) was performed with a flow rate of 1 mL per min. The mobile phase composition started with 100% solvent A, being increased linearly to 40% solvent B in 20 min, followed by a linear increase of solvent B to 60% in 10 min, and the final conditions being held for an additional 10 min. All samples were microfiltered before injection. The system was calibrated by standard compounds of (–)-epicatechin, (–)-epicatechin gallate, (–)-epigallocatechin, (–)-epigallocatechin gallate, and caffeine were purchased from Nagara Science. Stock standard solutions (500 ppm) were prepared in ascorbic acid and stored at −30°C, from which working standard solutions were prepared weekly by diluting with distilled water. Tea leaf extracts prepared as described above were analyzed according to the conditions for the calibration condition specified above.

### Statistical analysis

The data were statistically analyzed by Student’s *t*-test with one-sided and unpaired testing for unequal dispersion by using Microsoft Excel statistical tool. The level of significance in difference is defined as *p* < 0.01 and *p* < 0.05, specified in the legend for tables.

## Results

### Plasma lipid parameters

Basic feeding parameters of the study are presented in Table 1. The food intake record did not show a statistical difference in any feeding parameter amongst the feeding groups except for fat intake between the control group and the high fat-fed groups as designed and accordingly for the total energy intake as well. The major outcomes of the animal experiment after 8 weeks are presented in Table 2. No significant difference was found in the body weight among the groups at the end of the study while the relative weight of the visceral fat significantly decreased in the ST group against other groups (Table 3). The weight of the liver increased by HF feeding but significantly less in the ST group.

**TABLE 2:** Effects of 8 week tea-feeding; control chow (C), High fat chow (HF), Sichuan tea (ST), Pu-erh tea (PT), and Green tea (GT).

		C	High fat (HF)			
			
		Water	Water	ST	PT	GT
Body parameters	Body weight (BW), g	473.5 ± 22.3	507.1 ± 34.2	517.2 ± 31.2	552.8 ± 33.9	532.8 ± 28.9
	Liver/BW, %	2.90 ± 0.20	6.06 ± 0.72	5.39 ± 0.56^#^	5.63 ± 0.29	5.85 ± 0.45
	Visceral fat/BW, %	4.29 ± 0.60	4.64 ± 0.79	3.57 ± 0.65*	3.81 ± 0.76	4.35 0.67
	Cecal pH	7.32 ± 0.09	7.37 ± 0.18	7.08 ± 0.11*	7.26 ± 0.19	7.20 ± 0.17
Plasma parameters	Total cholesterol (C)	74.6 ± 12.0	82.1 ± 16.2	98.2 ± 16.4	108.2 ± 19.2*	100.5 ± 13.8*
(mg/dl)	HDL-C	43.0 ± 5.2	28.9 ± 3.8	37.0 ± 5.3**	40.5 ± 6.1**	31.6 ± 3.1
	non-HDL-C	22.8 ± 5.6	50.0 ± 13.5	63.9 ± 15.6	71.4 ± 19.8	69.6 ± 14.3*
	Triglyceride (TG)	71.0 ± 18.7	51.6 ± 17.8	36.0 ± 6.5*	52.0 ± 12.8	50.5 ± 10.6
	non-HDL-C/HDL-C	0.44 ± 0.07	1.56 ± 0.33	1.91 ± 0.51	1.96 ± 0.66	2.31 ± 0.61*
	TG/HDL-C	1.37 ± 0.33	1.58 ± 0.40	1.07 ± 0.16*	1.46 ± 0.49	1.68 ± 0.50
	Blood glucose	102.5 ± 15.5	131.4 ± 21.8	111.3 ± 14.0*	111.1 ± 8.2*	132.5 ± 18.3
mRNA in Liver	LXRα	9.2 ± 1.8	11.5 ± 2.8	23.7 ± 14.7*	12.8 ± 3.7	13.6 ± 4.1
(standardized by ß-actin)	PPARα	22.1 ± 9.7	29.6 ± 10.1	110.7 ± 100.6*	52.9 ± 44.1	23.5 ± 7.5
	PPARγ	77.8 ± 50.7	58.6 ± 32.4	151.5 ± 118.8*	101.2 ± 80.6	58.3 ± 27.8
	ABCA1	23.7 ± 4.5	28.7 ± 7.8	47.9 ± 19.5**	28.3 ± 8.0	31.2 ± 9.7
	ABCG1	171.3 ± 46.0	120.2 ± 29.4	102.3 ± 26.2	110.5 ± 39.3	94.1 ± 22.4
	Lipoprotein lipase	1.66 ± 0.77	1.73 ± 0.96	0.92 ± 0.36	1.31 ± 0.73	1.05 ± 0.40
	Hepatic lipase	0.91 ± 0.18	1.45 ± 0.56	1.93 ± 0.67	2.16 ± 0.47	2.37 ± 1.30

BW, body weight; liver and visceral fat,% to BW; BG, blood glucose. Asterisks indicate significant difference from HF without tea, as *p < 0.05 and **p < 0.01. ^#^Indicates *p* = 0.068 against HF without tea. For plasma lipid values, other significance parameters (*p*-values) are listed in Table 3.

**TABLE 3:** Significance of difference.

	HDL-C	non-HDL-C	TG	non-HDL-C/HDL-C	TG/HDL-C	Lipid deposit
C vs. HF	*p* < 0.01	*p* < 0.01	NS	*p* < 0.01	NS	*p* < 0.01
C vs. ST	*p* = 0.072	*p* < 0.01	*p* < 0.01	*p* < 0.01	*p* = 0.078	NS
C vs. PT	NS	*p* < 0.01	*p* < 0.05	*p* < 0.01	NS	*p* < 0.01
C vs. GT	*p* < 0.01	*p* < 0.01	*p* < 0.05	*p* < 0.01	NS	*p* < 0.01
HF vs. ST	*p* < 0.01	NS	*p* < 0.05	NS	*p* < 0.05	*p* < 0.01
HF vs. PT	*p* < 0.01	NS	NS	NS	NS	NS
HF vs. GT	NS	*p* < 0.05	NS	*p* < 0.05	NS	NS
ST vs. PT	NS	NS	*p* < 0.05	NS	NS	*p* < 0.01
ST vs. GT	*p* = 0.065	NS	*p* < 0.05	NS	*p* < 0.05	*p* < 0.01
PT vs. GT	*p* < 0.05	NS	NS	NS	NS	NS

**FIGURE 1:**
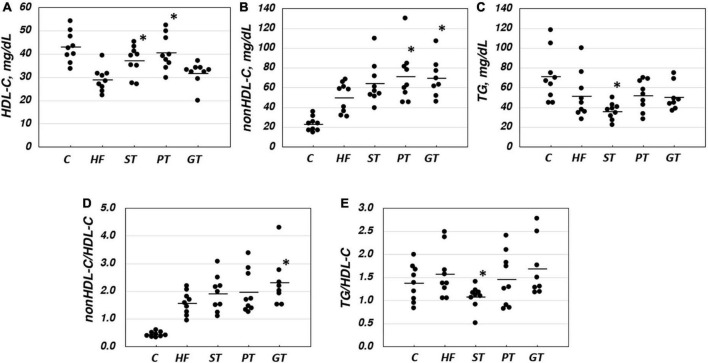
Lipid and lipoprotein parameters in rat plasma after 8-week experimental feeding as indicated in Tables 1, 2 (**A**, HDL-C; **B**, nonHDL-C; **C**, TG; **D**, nonHDL-C/HDL-C; **E**, TG/nonHDL-C) (C, control; HF, high fat-fed; ST, PT, and GT, Sichuan tea, Pu-erh tea, and Green tea on top of HF, respectively) (*n* = 9 for each group). A bar indicates the average value for each group. Total cholesterol and triglyceride (TG) were measured by using an enzymatic method and HDL-C was measured enzymatically by a homogeneous assay system as described in the text. Non-HDL-C was calculated by subtracting HDL-C from total cholesterol. Asterisks indicate a significant difference from the high-fat fed group (HF) as indicated in Table 3. The significance of differences in other combinations of the feeding groups is presented in Table 3.

**FIGURE 2:**
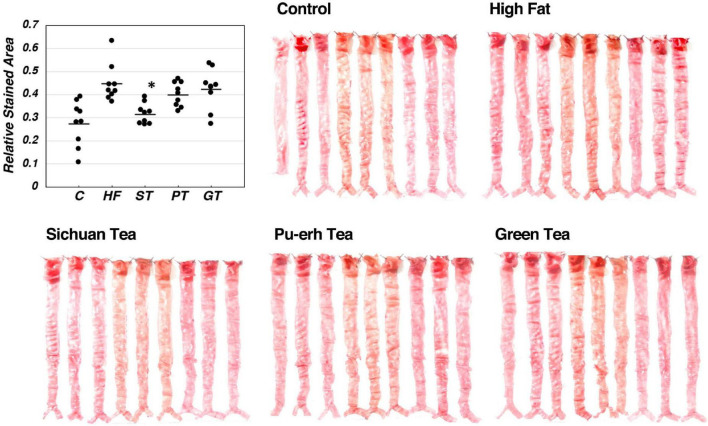
Lipid deposit on the aortic intima after 8-week experimental feeding, as indicated in Table 2 and Figure 1. The aortic intima surface was stained by Oil Red O. Red area was scanned and quantitated by using the Photoshop software as described in the text. The graph shows relative stained area and a bar indicates the average value for each group. The asterisk indicates a significant difference from HF as indicated in Table 3 (*n* = 9). The significance of differences in other combinations of the feeding groups is presented in Table 3.

The results of *t*-test by *p*-values for the data presented in Figures 1, 2, as control chow (C), High-fat chow (HF), Sichuan tea (ST), Pu-erh tea (PT), and Green tea (GT). NS, not significant.

Other abbreviations should be referred to Table 2.

Plasma lipid and lipoprotein levels at the end of the study are presented also in Table 2. Significance levels in the difference between the feeding groups are presented in Table 3 as *p*-values for each *t*-test combination. Significant changes by feeding HF diet were decreased in HDL-C and increased in non-HDL-C, but no apparent change was observed in TG level. Accordingly, non-HDL-C/HDL-C as a strong atherogenic index showed a marked increase indicating an increase in atherogenesis in this rat model. On the basis of HF diet feeding, HDL-C significantly increased in the ST and PT groups against HF but the GT group showed no difference. Non-HDL-C increased significantly by PT and GT against HF. TG decreased by ST against other groups fed with HF. The parameters of non-HDL-C/HDL-C increased by GT against HF and TG/HDL-C decreased by ST against HF, PT, and GT. Blood glucose decreased in ST and PT but not in GT groups against the HF group. Plasma lipid data are also graphically displayed in Figure 1. The peculiar changes by feeding of ST were demonstrated as an increase in HDL-C and a decrease in TG and accordingly a decrease in TG/HDL-C, in comparison with other tea products.

### Expression of the lipid-related genes

Table 2 also shows the data for expression levels of some mRNAs related to lipid and lipoprotein metabolism probed in the liver of the rats after the 8-week experiment. The mRNA of LXRα, PPARα, PPARγ, and ABCA1 was increased by ST against other feeding groups, but the expression of ABCG1, lipoprotein lipase, and hepatic lipase showed no significant difference among the groups. Overall, the anti-atherogenic effect of ST was more prominent than PT and GT (Table 3).

### Lipid deposition on the aorta

Lipid deposition in the aortic intima surface was visualized by Oil red O staining after feeding of the tea preparations for 8 weeks, as shown in Figure 2. The area of the stained region relative to the total aortic surface was calculated for each animal and displayed in the graph in the figure. Significance between the feeding groups is presented in Table 3 as the *p*-values of each *t*-test between the groups. Lipid deposit significantly increased by HF diet indicating atherogenesis of this diet model, and it was decreased by feeding ST. The feeding groups of PT and GT did not show a significant change in lipid deposit.

### Analysis of the tea extracts and their effects on the cells in culture

**FIGURE 3:**
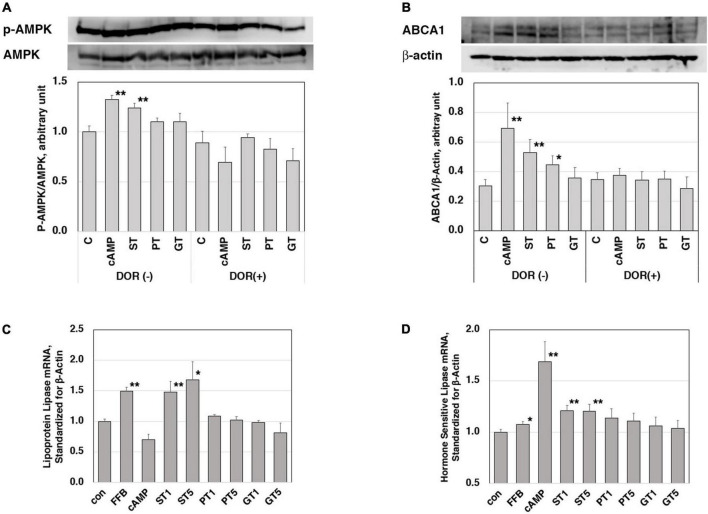
Functions of the tea extracts evaluated in the cell culture system. Stimulation of AMPK phosphorylation in mouse macrophage cell line cells J774 **(A,B)**. Cells were pre-incubated with 5 μM AMPK inhibitor dorsomorphin (DOR) for 1 h and then incubated with or without 0.15 mM cAMP or tea extracts (1 mg/ml) for 4 h. AMPK protein, phosphorylated AMPK **(A)**, and ABCA1 **(B)** were quantitated in electrophoresis and immunoblotting. Relative phosphorylation was calculated for graphic display (C for control; cAMP as a positive control; ST, PT, and GT for Sichuan tea, Pu-erh tea, and Green tea, respectively). Asterisks ** indicate significant differences from the control by *p* < 0.01 (*n* = 3). Enhancement of expression of lipoprotein lipase **(C)** and hormone-sensitive lipase **(D)** in mouse adipose cell models 3T3-L1. Cells were stimulated by tea extracts (1 and 5 μg/ml), fenofibric acid (50 μM), and cAMP (0.15 mM). The mRNA of lipoprotein lipase and hormone-sensitive lipase was quantitated by RT-PCR (C for control; FFB, fenofibric acid; ST, PT, and GT for Sichuan tea, Pu-erh tea, and Green tea, respectively). Asterisks indicate significant differences from the control by **p < 0.01 and *p < 0.05. The data presented represent those from 5 to 6 experiments repeated.

The increase by ST in mRNA of LXRα, PPARα, PPARγ, and ABCA1 in the rat liver was consistent with the increase in their plasma HDL. In order to investigate a signal to trigger these reactions, stimulation of the AMPK pathway was examined in J774 mouse macrophage cell line cells (Figures 3A,B). A positive control cAMP induced phosphorylation of AMPK as well as an increase of ABCA1 in this system and these effects were canceled by an AMPK inhibitor DOR. The extract from ST also significantly induced phosphorylation of AMPK but no other tea extracts, as well as an increase in the expression of ABCA1. The effects of ST were also canceled by DOR. Thus, AMPK phosphorylation is likely an upper-stream signal for LXRα and PPARs to increase ABCA1 gene expression by ST similar to the case demonstrated with nobiletin (23).

Reduction of plasma TG and body fat mass seem to be the other key for the anti-atherogenic effects of ST, so the molecular background for these functions was examined in mouse adipose-like cells 3T3-L1. The expression levels of mRNA of lipases were examined for the effects of the tea extracts (Figures 3C,D). The mRNA of lipoprotein lipase and hormone-sensitive lipase was increased by the respective positive control activator of fenofibrate (PPARα agonist) and cAMP. Among the three tea extracts, ST increased the mRNA of both lipases being consistent with the phenotype induced by ST in fat-fed rats.

Profile of representative bioactive components, such as caffeine and catechins of tea extracts were analyzed by HPLC. Substantial differences were demonstrated in the spectrum of caffeine and four typical catechins (such as epigallocatechin, epicatechin, epigallocatechin gallate, and epicatechin) among the tea preparations examined (Table 4). However, none of those differences were likely consistent to support the specific effects of ST, including the change in caffeine which most likely stimulates the cAMP-related signaling pathway.

**TABLE 4:** HPLC analysis of tea extract.

	Caffeine	Catechin			
		
		EGC	EC	EGCG	ECG
ST	738	<31	<11	380	102
PT	690	<13	108	<4	44
GT	491	720	65	853	160

EGC, epigallocatechin; EC, epicatechin; EGCG, epigallocatechin gallate; ECG, epicatechin gallate, as ppm in tea preparation.

## Discussion

Sichuan dark tea, a type of tea produced by fermentation in a specific traditional fashion in China, has been claimed to have various beneficial effects against lifestyle-related health disorders, such as diabetes, dyslipidemia, and atherosclerosis as described above. However, solid experimental or clinical evidence have not adequately accumulated to support these claims (12–14). A recent report indicated that ST-related product induced a reduction in TG and an increase of HDL in apolipoprotein E-deficient mice as well as changing the intestinal flora (15). We, therefore, conducted experiments to investigate the effects of ST including verification of the prior findings reported and to look into their molecular background. The atherogenesis model was generated by a modest increase of fat feeding in order to reflect the Japanese nutritional background (18–20). This diet resulted in a significant decrease in HDL-C and an increase in non-HDL-C and accordingly marked an increase in the atherogenic index of non-HDL-C/HDL-C ratio, without apparent change in TG. Lipid deposit in the aortic surface indeed increased by this diet feeding.

Sichuan dark tea feeding increased HDL and reduced plasma TG in rats to confirm the previous findings in mice (15). Both of these animals lack plasma cholesteryl ester transfer protein activity, so these findings are independent effects and unlikely linked (25). ST also induced a significant decrease in visceral fat and blood glucose level. Accordingly, ST reduced fat deposits on the aortic intima. Thus, the potential anti-atherogenic effect of ST was demonstrated likely through altering lipid and lipoprotein metabolism. ST reduced the cecal pH, which may also be consistent with the previous finding of changing the intestinal flora. Pu-erh tea (PT) and GT, shown effective in improving lipid metabolism and atherosclerosis in model animals with severe hyperlipidemia (17, 26–28), were less effective in a current moderate hyperlipidemic rat model.

Plasma HDL level and lipid accumulation in vascular macrophages are regulated by the expression of ABCA1, and its mRNA indeed increased in the liver of ST-fed rats being consistent with the finding in the animals. We previously showed that regulation of ABCA1 expression involves the cAMP-AMPK pathway (29) to activate the propagation loop between LXRα-PPARs (23, 30). AMPK was shown activated by ST to increase ABCA1 expression in a macrophage cell model to support the finding that ST decreased lipid deposition in the aorta. Reduction of plasma TG and body fat mass can also be related to the cAMP-AMPK pathway. The increase of PPARs upregulates LPL expression to reduce plasma TG. The cAMP pathway also increases the expression of hormone-sensitive lipase to mobilize TG from adipocytes to decrease body fat. These hypotheses were consistent with the findings demonstrated in an adipose cell model as the increase of mRNAs of these lipases by the ST extract. Thus, the effect of ST on lipid metabolism can largely be interpreted by its stimulation of the cAMP-AMPK pathway, which eventually achieves the prevention of lipid deposition on the aortic surface. This mechanism has been in fact suggested as a background for anti-atherogenic effects of other teas (31–33).

It was inconclusive at this moment to identify a specific substance for this function of ST among these known tea compounds. Well-known active components in teas are caffeine and catechins, which potentially activate the cAMP-AMPK pathway (34–37). These components are analyzed by HPLC for the extract of the teas used in this study. There was a substantial difference in their composition among ST, PT, and GT, but no specific tendency was identified to interpret or support the specific anti-atherogenic effect of ST. It has been demonstrated various modifications and bioconversion of the authentic bioactive components of tea leaves during their processing by fermentation (38–43). Specific fungal and bacterial spectrums required for fermentation to produce ST make such reactions more complicated (44, 45).

We thus conclude that ST may be more beneficial for the prevention of atherosclerosis development than other types of tea, at least PT and GT, by regularly taking it in a normal beverage-taking fashion. Investigation into the profile of plasma lipoprotein and apolipoprotein in future would provide more supportive information for the hypothesis above discussed. Further study is required to identify a specific compound(s) or any specific combination of such materials contained in ST for its peculiar anti-atherogenic effects. The role of low cecal pH in ST-fed rats is unknown if it is related to the effects on lipid metabolism or more directly to anti-atherosclerotic action. This should be investigated in relation to changes in the intestinal flora by the ST-related product (15).

## Conclusion

**FIGURE 4:**
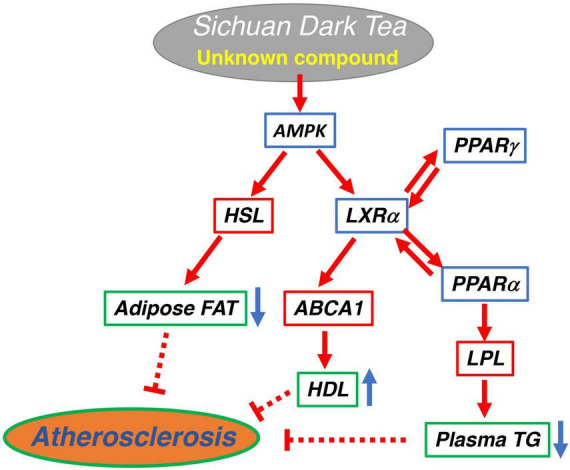
Graphical summary of the potential signaling pathway for anti-atherogenic effect of Sichuan dark tea.

Sichuan dark tea increased HDL and reduced plasma TG and visceral fat and decreased lipid deposition in the aorta in fat-fed rats, more than Pu-erh tea and Japanese green tea. ST increases ABCA1, lipoprotein lipase, and hormone-sensitive lipase by activation of the AMPK pathway. The results and discussion are graphically summarized in Figure 4. The effect however cannot be attributed to caffeine and catechins in their original forms. Further studies are required to identify the compounds responsible for this specific function.

## Data availability statement

The raw data supporting the conclusions of this article will be made available by the authors, without undue reservation.

## Ethics statement

This animal study was reviewed and approved by the Animal Experiments and Welfare Committee of Chubu University.

## Author contributions

RL initiated the project, designed the experiments, carried them out herself, analyzed the data, and wrote the manuscript. TSu performed animal experiments for his master-degree graduate study. TT assisted the animal experiments and helped cellular experiments *in vitro* with her expertise. TSe contributed to animal experiments and cell culture mainly collaborating with TSu. MT helped animal experiment in evaluating intestinal physiology. XL provided the basic idea for the project on Sichuan black tea and contributed to obtaining the materials. SY supervised designing the projects and experiments, analyzing the data, and writing the manuscript. All authors contributed to the article and approved the submitted version.

## Abbreviations

ST: Sichuan dark tea
PT: Pu-erh tea
GT: Japanese green tea
HDL: high density lipoprotein
LXR: liver X receptor
PPAR: peroxisome proliferator-activated receptor
ABC: ATP-binding cassette transporter
HF: high fat diet group
TC: total cholesterol
HDL-C: HDL-cholesterol
non-HDL-C: non-HDL-cholesterol
TG: triglyceride
AMPK: 5 ′ adenosine monophosphate-activated protein kinase
DOR: dorsomorphin
SDS: sodium dodecylsulfate
HPLC: high performance liquid chromatography.
